# Revealing urban area from mobile positioning data

**DOI:** 10.1038/s41598-024-82006-5

**Published:** 2024-12-28

**Authors:** Gergő Pintér

**Affiliations:** ANETI Lab, Corvinus Institute for Advanced Studies, Corvinus University of Budapest, Budapest, 1093 Hungary

**Keywords:** Mobile positioning data, Urban mobility, YJMob100K, HuMob2023 challenge, Reverse-engineering, Computer science, Scientific data

## Abstract

Researchers face the trade-off between publishing mobility data along with their papers while protecting the privacy of the individuals. In addition to the anonymization process, other techniques, such as spatial discretization and location concealing or removal, are applied to achieve these dual objectives. The primary research question is whether concealing the observation area is an adequate form of protection or whether human mobility patterns in urban areas are inherently revealing of location. The characteristics of the mobility data, such as the number of activity records in a given spatial unit, can reveal the silhouette of the urban landscape, which can be used to infer the identity of the city in question. The presented locating method was tested on multiple cities using different open datasets and coarser spatial discretization units. While publishing mobility data is essential for research, concealing the observation area is insufficient to prevent the identification of the urban area. Instead of obscuring the observation area, noise should be added to the trajectories to mitigate privacy risks regarding the individuals.

## Introduction

In order to ensure the reproducibility of research findings, it is advisable for scholars to publish both data and code alongside their papers. Nevertheless, the publication of mobility data raises issues regarding privacy, as the process of anonymization is complex and challenging. It has been demonstrated on numerous occasions that the implemented anonymization process was inadequate. The users in the Netflix Prize dataset were re-identified using IMDb as a source of background knowledge^1^. The taxi IDs of the NYC taxi data were also re-identified, revealing the drivers’ identity due to poor anonymization technique^2^. In combination with paparazzi photographs from social media, which often capture the unique IDs of the cabs, celebrities can be tracked in the NYC dataset^3^. Similarly, the ticket IDs in the published data of the Riga public transport were easily de-anonymized^4^, revealing the ticket type, which could lead to privacy attacks.

Mobile positioning data can be affected as well. As demonstrated by Sharad and Danezis in their analysis of the anonymization of the D4D challenge data^5^, was found to be inadequate^6^. Even if the anonymization process was performed correctly, and the user is not identifiable by any columns, the visited locations still provide some attack vectors based on the individuals’ behavior. De Montjoye et al. showed that the vast majority of the individuals could be distinguished from each other by only four spatial data points^7^. Another prevalent technique is spatial discretization^8–10^, which can limit the accuracy of geographic locations to a certain level.

This paper presents evidence that concealing the observation area of mobility data cannot be an effective solution to privacy concerns, because human activity are tightly connected to the urban areas, and it will reveal the observation area. To achieve this, I primarily use the ‘YJMob100K’ data set^11^, a metropolitan-scale, longitudinal, anonymized mobility trajectory data set that aims to serve as a benchmark dataset of human mobility^12^. The data provider is Yahoo Japan Corporation, and the data follows 100,000 individuals across a 90-day period in an undisclosed, highly populated metropolitan area in Japan^11^. In this data set the geographic locations of the individuals are discretized (into 500-meter by 500-meter cells), and the location of the observation area is undisclosed. Furthermore, the precise dates are not provided; instead, the days are numbered relatively (e.g., day 1, day 2, etc., up to day 90) and the time is also discretized into 30-minute intervals.

The data has been discretized both spatially and temporally, and user IDs are sequential numbers. However, the main question is whether the undisclosed observation area provides sufficient protection. Alternatively, the characteristics of human mobility can reveal in which urban area the mobility data was captured. Despite the absence of geographic locations for the spatial grid cells, the activity distribution within the grid can be used to infer the city landscape. Once the urban area was deduced using a map, a template matching method was applied to find the exact grid location on the map, which is a technique in digital image processing for finding the location of a template image in a larger image. The spatial grid used to discretize the mobility data can be reconstructed once the location of the observation area is known.

The presented approach was generalized in two directions. Initially, coarser discretization resolutions were simulated, demonstrating that city characteristics cannot be easily suppressed. Second, the location technique, based on template matching, was presented for four other cities from three additional data sets. Furthermore, in one of these cases, H3 hexagons were used instead of a grid, indicating that the discretization scheme is also irrelevant.

The majority of human activity takes place in urban environments. Therefore, if a sufficient amount of location data is available, it will inherently reveal the city in which the data was collected. By itself, the revealed observation area does not pose a threat to the privacy of individuals. However, it does make it easier to link other data sources to the mobility data for more detailed profiling. Estimation of home (and work) location estimation is a common part of the mobility data processing^13–15^, such as the income proxying by the real estate prices of the home location^16,17^. A possible attack vector is to look for people in the mobility data whose appearances are well known, such as celebrities or politicians, and use this information to infer which user is the given person. For example, the President of the United States^18^. The bottom line is that privacy by obscurity is not viable solution for ensuring true privacy. The privacy-preserving transformation applied to the mobility data should not depend on an obscured dimension of the data. Previous studies have shown that adding noise to the location trajectories can mitigate privacy issues^2,19,20^, when synthetic mobility data is not a viable option. In addition, the spatial dimension can be omitted completely from mobility data^21^.

## Results

The urban area in which the activities were recorded was determined by analyzing the visible silhouette of the urban landscape in the activity heatmap^11^, Figure 6, and assuming that the low-activity areas are partly water surfaces. The urban area was identified as the Nagoya metropolitan area including Mikawa Bay and Ise Bay. Subsequently, the spatial grid, which was used to discretize the geographic locations of the individuals, was reconstructed. This section presents a mobility analysis, which serves as a validation of the grid reconstruction. The reverse-engineering process is detailed in the Methods section.

**Fig. 1:**
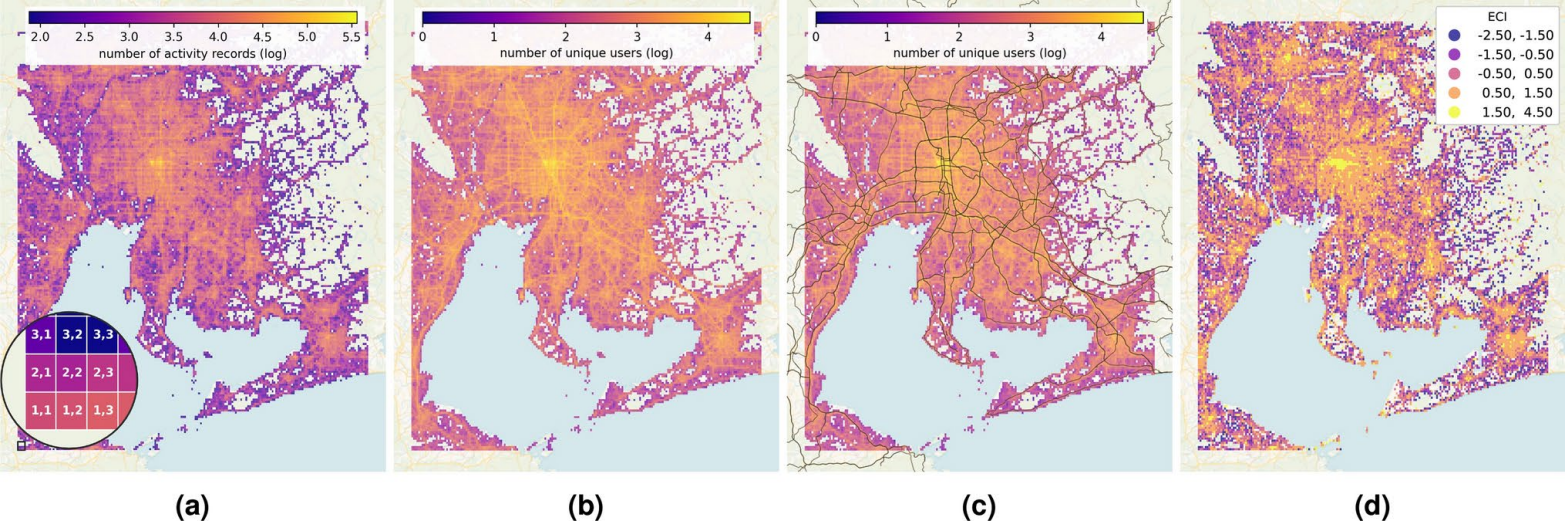
The reconstructed grid is plotted over a map, with colors indicating the number of activity records (**a**) and unique users (**b**) on a log-scale, as well as the higher-order elements of the road network were also displayed (**c**) , and the amenity complexity (**d**) of the cells for additional details. To highlight the coastline, the cells were set to transparent if the activity value is below the same threshold as for Figure 7c. Generated with own code, available on GitHub.

The first step in the validation process is to plot the reconstructed grid over the map, with each cell colored according to the number of activity records (Figure 1a) and unique users (Figure 1b). The number of unique users per cell more accurately reflects the road network, particularly the highest order of highways (motorway, trunk) from the OSM, which were displayed as a validation (Figure 1c). As the grid aligns with the map, it can be concluded that the grid geometry is considered good.

The amenity complexity, as defined by Juhász et al.^22^, can be calculated using the provided the information about the number of different POIs (POI) in each cell, which is provided for the YJMob100K data. The POIs are also anonymized otherwise it would help to locate the grid on the map. Only the cardinality of each anonymized category is provided in the YJMob100K dataset, which is sufficient to calculate the amenity complexity. Complex amenities attract socially diverse people^22^ and can indicate city centers. It is used in this paper as a part of the validation process, with the expectation that the highest values are clustered around the city centers. Figure 1d shows the Economic Complexity Index (ECI) in terms of amenities, and the highest ECI values indeed correspond to downtown Nagoya and the center of other cities.

Another direction of the grid validation could be the home detection. It is common practice^13–15^ to apply home detection algorithms to mobility data. In this case, the cell with the most activity after 21:00 and before 8:00 is considered the home location. It should be noted that the home detection does not require the reconstructed grid it can be computed from the original data. The thresholds (21:00 and 8:00) are set as a rule of thumb and may require adjustment to align with Japanese societal norms. Furthermore, the home detection algorithm does not differentiate between workdays and weekends as the data does not contain dates. However, holidays could be inferred from the daily activity levels.

**Fig. 2:**
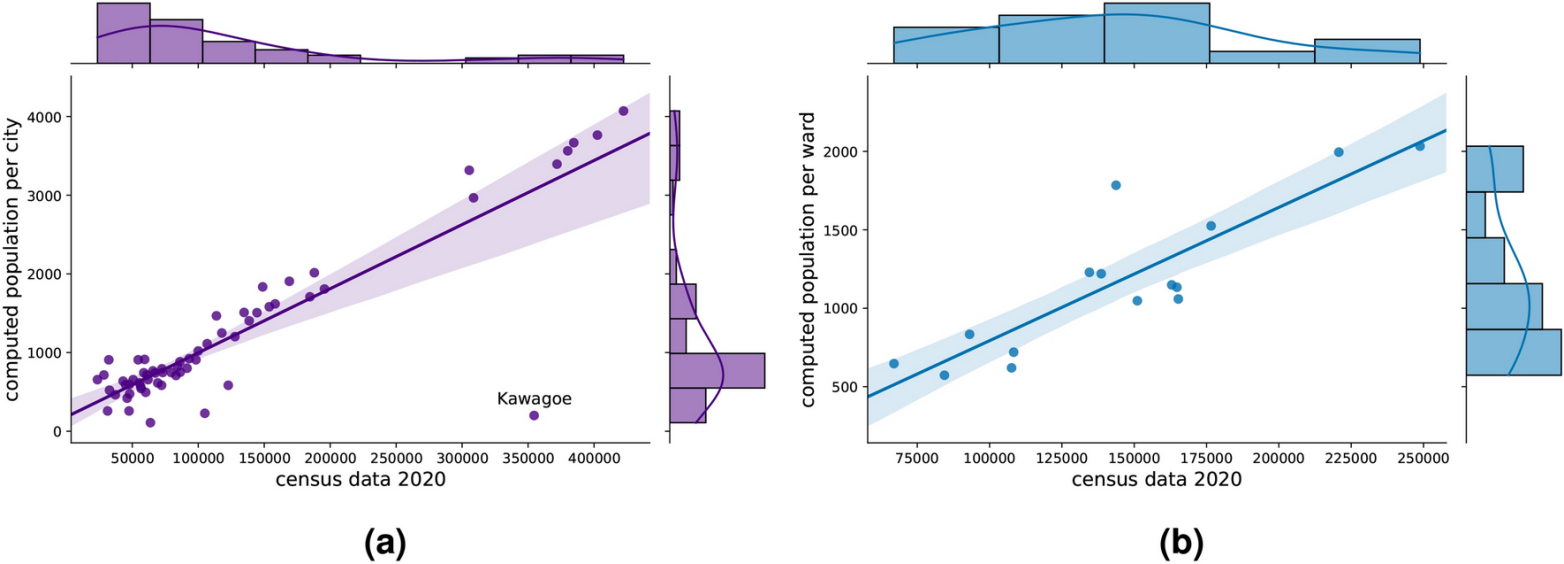
The correlation between the population based on census data and the estimated number of inhabitants. The comparison is made at the municipal level (**a**) and also on the ward level of Nagoya (**b**). Cities whose area is covered by the observation area with less than 30% are excluded from the comparison.

Without ground truth, the detected home locations cannot be validated, but their spatial distribution can be compared^22,23^to the census data^24^. Figure 2a compares the estimated number of inhabitants for the cities within the observation area with the 2020 census data. The Pearson correlation coefficient is 0.8879. It should be noted that cities with less than 30% of their area within the observation area have been excluded from the comparison. Interestingly, Kawagoe is still an outlier (denoted in Figure 2a) and appears to be underrepresented in the mobility data. Upon removing Kawagoe from the comparison, the city-level Pearson correlation coefficient increases to 0.9717. The results show that the data follows approximately 1% of the population. Figure 2b shows the correlation at the ward level in Nagoya (correlation coefficient is 0.8744).

The spatial distribution of the detected population correlates with the census data, thereby confirming the validity of the constructed grid geometry. In contrast, the correlations are notably worse in the case of the unstretched grid (Figure 7f), where Pearson correlation coefficient is 0.7536 for the municipality level and 0.4676 for the ward level.

### Robustness

**Fig. 3:**
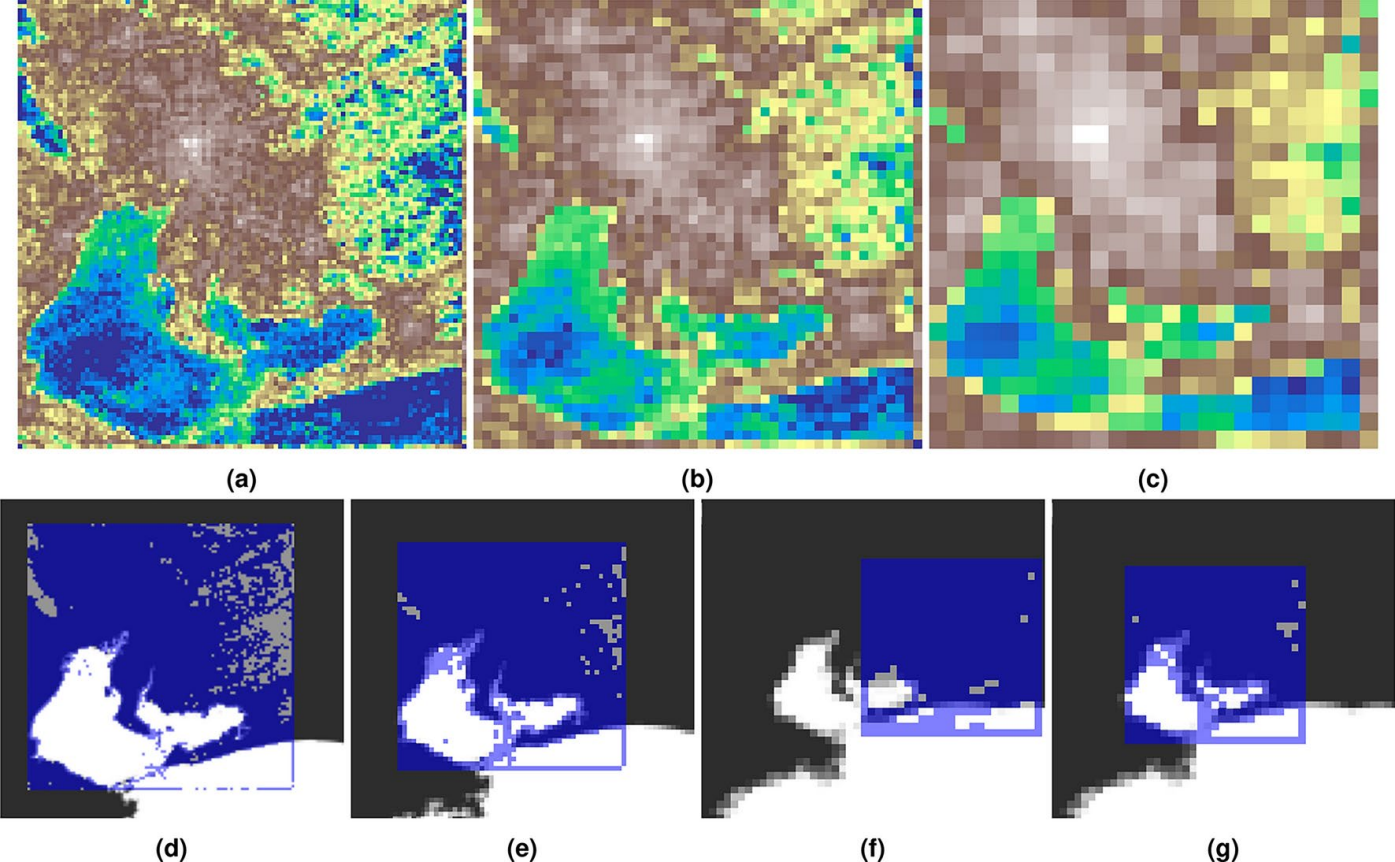
Locating the upscaled grids. The upper row shows the upscaled grids plotted as heatmaps. Instead of the original 500 m by 500 m grid, a 1 km by 1 km (**a**), a 2 km by 2 km (**b**), and a 4 km by 4 km (**c**) cells are used, resulting $100\times 100$, $50\times 50$ and $25\times 25$ element matrices. The second row shows the results of the template matching for the upscaled grids. In the first two cases the locating succeeded using the original template threshold (75) (**d**, **e**), but failed for the 4 km by 4 km case (**f**). Therefor, the threshold was increased to 375 succeeding the template matching for the 4 km by 4 km grid as well (**g**).

It was previously demonstrated that the urban landscape can be inferred from mobility data even if the observation area is undisclosed. To what extent is the presented method robust to increases in the resolution of the discretization? The reconstructed grid was merged into grids of 1 km by 1 km, 2 km by 2 km, and 4 km by 4 km grids by summing the activity records in 4, 16, and 64 cells, respectively. An increase in the cell size results in a reduction in the accuracy of user location, thereby increasing privacy. The first row of the Figure 3 shows the upscaled grids as heatmaps using the same color scheme as in Figure 6d. Although the rescaled grids notably compress the information and the figures lose details, the urban landscape remains more or less recognizable. The expectation is that the presented approach, utilizing template matching, will be able of accurately identifying the urban area.

The template was generated with the same threshold (75) as for the original grid (Figure 7c). Second row of the Figure 3 shows the location results. In the first two cases (Figure 3d and 3e), the locating was accurate. However, it failed for the 4 km by 4 km grid (Figure 3f). The threshold proved too low concerning the compressed image as the shape of Mikawa and Ise bays was lost, so the threshold was increased to 375. Upon increasing the threshold, the template matching algorithm was able to successfully locate the urban area (Figure 3g). This result proves that the characteristics of human mobility can reveal the urban landscape even when the mobility locations are discretized into 4 km by 4 km cells.

Another direction of the robustness checks is extending the presented method to other cities. The openly available Weeplaces data set^25^was used, presented in^26^, which was collected from LBSN including Facebook Places, Foursquare, and Gowalla. Two cities were selected from the Weeplaces database for further analysis: Toronto and London. The activity locations were discretized into a 500 m by 500 m grid over a 100 km by 100 km by area, which also covers other settlements from the area similarly as in the YJMob100K data set^27^. Note that an area of this size covers other settlements outside Toronto from the shore of Lake Ontario. The Weeplaces data set contains a significantly lower number of activity records per city than the YJMob100K data set. In the case of Toronto, there are 110,829 activity records from 19,356 unique users in the selected area, whereas the YJMob100K data set contains 111,535,17 activity records from 100,000 users.

**Fig. 4:**
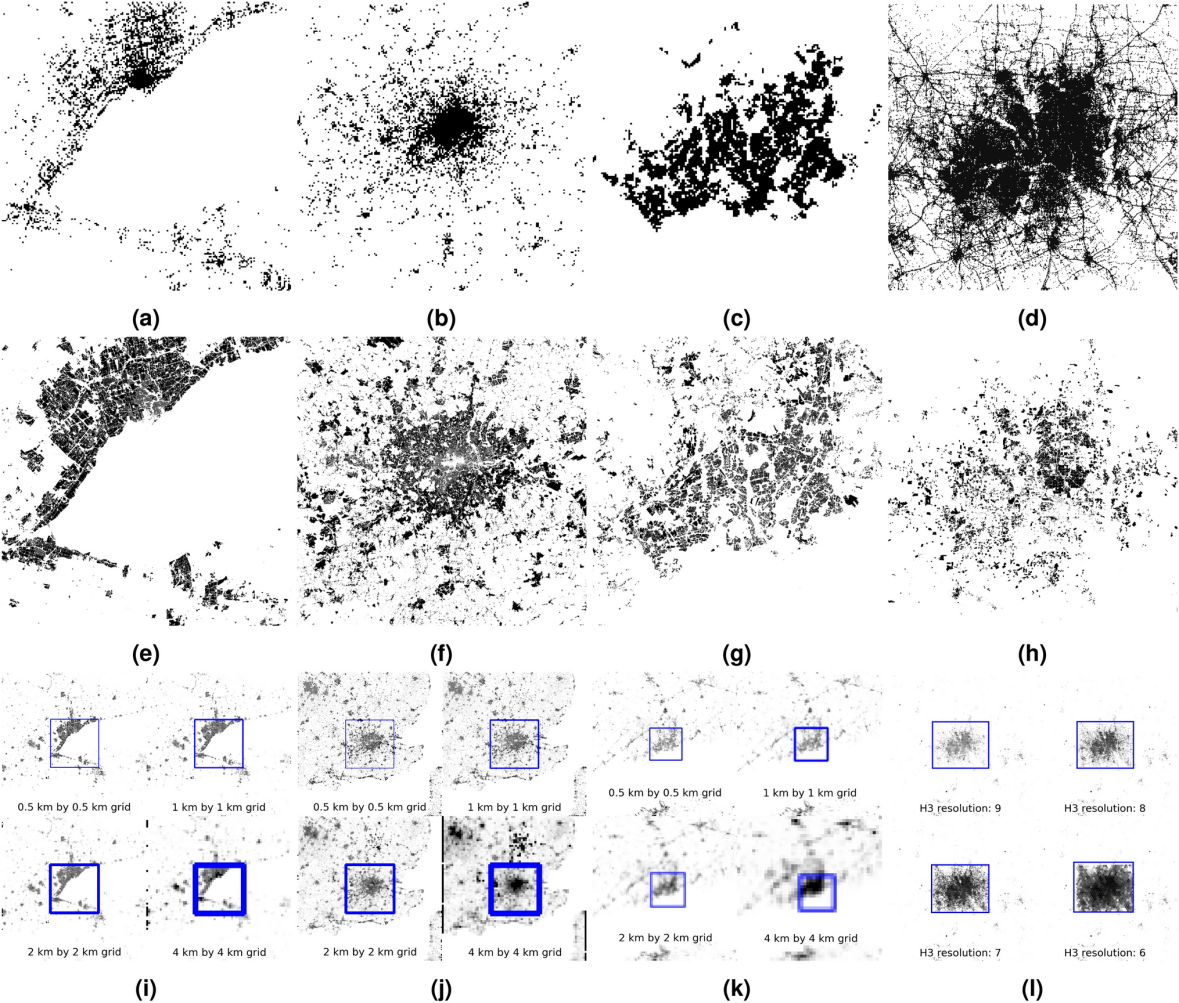
User activity from the Weeplaces data set discretized into a 500 m by 500 m grid around Toronto (**a**), London (**b**), Helsinki with a 250 m by 250 m grid from the population distribution data set (**c**), and pings from the Dallas–Fort Worth metroplex (**d**). The residential, retail, and industrial areas extracted from the OSM (**e**, **f**, **g**, and **h**, respectively). The located urban areas of Toronto (**i**), London (**j**) and Helsinki (**k**), using 500 m, 1 km, 2 km and 4 km squares, and H3 hexagons at resolutions of 9, 8, 7 and 6 for the Dallas–Fort Worth metroplex (**l**) as the discretization method.

The Weeplaces data set is notable sparser, with significantly less activity concentrated outside the urban areas (Figure 4a), in contrast to the YJMob100K data set, where unpopulated areas still have some activity, even the ferry lines can be recognized. The sparseness of the available data for Toronto makes it more difficult to compare the activity heatmap to the land. Furthermore, cities without distinctive coastal regions, such as London (Figure 4b), may not work. Alternatively, land usage data was extracted from OSM, namely regions designated as residential, retail, or industrial (Figure 4e and 4f). This approach aligns with the functional classification of the urban areas with respect to land cover data presented in^9^. In this case, the template image contains black if the cell has any activity and white otherwise. Figure 4i and 4j illustrate that the urban area can be located using 500 m, 1 km, 2 km, and 4 km squares using the same method as in the case of the Nagoya metropolitan area (Figure 3).

A temporal density dataset was published^9^ from the Helsinki Metropolitan Area. It uses a 250 m by 250 m grid for the spatial dimension, which contains the portion of the population that was present in a given cell by hours. For the template, the distribution values from the workday table at 21:00 were used, and the threshold value was 0.0025 (Figure4c). The template was compared to the land usage data from OSM (Figure 4g). Figure 4k shows the result of the template matching using 500 m, 1 km, 2 km, and 4 km grids.

Another data set was used to demonstrate the city landscape recognizability, covering the Dallas–Fort Worth metroplex^28^from the paper^29^, which contains hourly locations of people (Figure 4d). As opposed to the previous procedure, the coordinates are discretized using H3 hexagons^30^, and the activity aggregated to different sizes of H3 hexagons are compared to the land usage (Figure 4h). The H3 resolutions between 6 and 9 are tested. Larger resolutions represent smaller hexagons: a resolution-9 hexagon is about 0.1053 km^2^, a resolution-8 hexagon is about 0.7373 km^2^, a resolution-7 hexagon is about 5.1613 km^2^, and a resolution-6 hexagon is about 36.129 km^2^. The template matching algorithm determined the city location well in the three higher resolution cases, but slightly misplaced the template when using the resolution-6 hexagons (Figure 4l).

### User traceability

It is worth testing how the user traceability changed by changing grid scales. A user is traceable, stands out from the crowd, if the top four locations are distinguishable^7^. For the traceability test, the 1 km, 2 km, 4 km, 8 km, and 16 km square grids were used. The activities were remapped to the upscaled grids, and the top four locations were determined. A user is considered traceable in the upscaled grid if the most visited four locations can still be distinguished. Table 1 shows the number of traceable users by five upscaled grids. More than 35% of the users are still traceable using the 1 km by 1 km grid as the original top four locations are still distinguishable. 5% of the users are still traceable using the 4 km by 4 km grid, which also made the observation area recognizable (Figure 3g).

**Table 1:** Comparison of the top-four-location traceable users by upscaled grids.

Distinguishable cells	1 km x 1 km	2 km x 2 km	4 km x 4 km	8 km x 8 km	16 km x 16 km
4	35469	12882	5090	1810	470
3	48228	42323	28457	16752	7438
2	15582	38548	50987	52608	44939
1	721	6247	15466	28830	47153

## Discussion

The observation area of the mobile positioning data sets is usually communicated. However, a recently published data set^11^, did not disclose the geographic location of the mobility traces. This study examines how effective this step is in terms of user privacy. The main finding is that mobile positioning datasets describe the urban landscapes where the mobility took place, so the geographic location can be identified even if it is not disclosed. In addition to the ‘YJMob100K’ data set, this was demonstrated in four other cities from three other openly available data sets. Furthermore, this effect has some resilience to upscaling, so that the urban area can be identified even with lower resolution grids.

It is important to emphasize that the goal of this work is not to demonstrate template matching on mobility data but to point out that human activity has a peculiar spatial distribution that makes the urban landscape recognizable even after discretization. With the uncovered metropolitan area, it is now possible to cluster the users based on home location (on a city, a ward, or even a neighborhood level), which information can be associated with real estate prices to profile mobility based on estimated income (e.g., as^16,17^ uses data from the Japanese statistical office). The revealed geographic location widen the possible applications of the YJMob100K data but at the same time it might increase privacy risks by making it easier to link additional data sources. Furthermore, may provide possibility to look for people in the mobility data whose appearances are well known, and use this information to infer which user could be the given person.

In cryptography, it is widely accepted that there is no security by obscurity^31^. Based on this principle, privacy through obscurity could not provide privacy. However, in this case, the obscured observation area was meant to provide another layer of protection in addition to the anonymization, and the spatial and temporal discretization. Zhang and Bolot argue that publishing location data is likely to lead to privacy risks, and the data must be coarse in either the time domain or the space domain^32^. Even partial location data can be used to infer the location. In^33^, the altitude information from fitness tracker applications was used to infer the location of users.

This discourse leads to a trade-off between privacy preservation and researchers’ interest in using more granular mobile positioning data to build better models. In addition to adding noise to the location data^19^, a possible solution for protecting user privacy when publishing mobility data is to exclude location information completely. For example, a mobility data set was published^21^, covering Changchun Municipality, Northeast China, revealing only distances between locations. In Section S1 of the supplementary information, it was shown that the urban landscape remains recognizable even if each location coordinate is transformed by adding random noise. Adding noise to (a part of) the locations, or adding fake appearances can mitigate attacks on user identification based on visitation patterns (e.g^18^.,).

This work is not without limitations. First, the reconstructed grid cannot be arbitrarily accurate. The accuracy of the grid anchor point is at most 500 meters in both directions (using the original resolution), because the template matching returns the coordinate of a pixel that represents a 500-meter by 500-meter cell. As with the upscaled grids, the larger the grid size, the greater the chance that the anchor point will be misplaced. The users’ geographic locations are discretized into 500-meter by 500-meter grid cells, so the accuracy is inherently limited, but the inaccuracy of the reconstructed grid placement increases it further. On the other hand, the presented validation process with the population counts at the ward and municipality level shows that the determined location of the grid is acceptable.

Note that the template matching was applied to the area surrounding the selected city with a limited size instead of the whole globe, which could be possible with sufficient computing capacity. Furthermore, the applied template matching solution cannot deal with scaling or rotation (it was not necessary), although there are rotation and scale invariant template matching algorithms (e.g^34^.,).

Originally, the visibility of the road network in the heatmap^11^ gave the idea that the urban landscape can be identified from mobility data, but using the road network with the presented template matching approach did not give satisfactory results. This may be mainly because the roads are much narrower than the grid cells. Also, the activity around the roads is not necessarily distributed proportionally to the priority or the order of a road. For example, a highway is considered higher order than a street in a shopping district, but the latter may contain more user activity. Thus, it is difficult to plot a map based solely on the cartographic features of the roads in a way that would be matched with the activity-weighted and spatially distorted templates. For these reasons, the road network-based template matching could not match the landscape-based approach with the algorithm used. However, this does not mean that only binary templates could work as it was demonstrated in Section S2 of the supplementary information.

The initial hypothesis of this study was that mobility trajectories can identify the urban area where the mobility took place, even if the location is not disclosed. This hypothesis was tested on the ‘YJMob100K’ data set, and is considered confirmed with the reconstructed grid. The results show that hiding the observation area does not provide significant privacy benefits. It was also shown that coarser grids can reveal the observation area.

## Methods

**Fig. 5:**
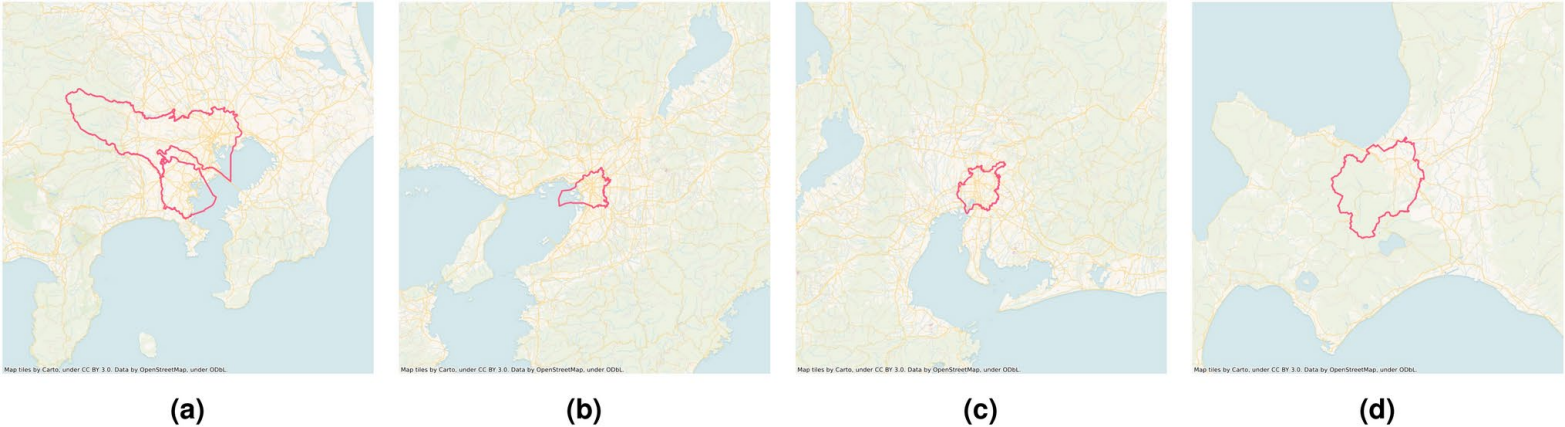
The administrative boundaries of Japan’s five largest cities: Tokyo (**a** top), Yokohama (**a** bottom), Osaka (b), Nagoya (c) and Sapporo (d). Generated with own code, available on GitHub.

Two pieces of information was required to deduce which city is in the focus of the data: (i) it was revealed that the metropolitan area is in Japan, and (ii) the urban area must be large as the observation area is 100 km x 100 km. Taking into consideration the five largest cities of Japan^35^ (Figure 5), the observation area might be Tokyo, Yokohama, Osaka, Nagoya or Sapporo, which are all near the sea (or ocean), so the low-activity parts might be waterfaces.

**Fig. 6:**
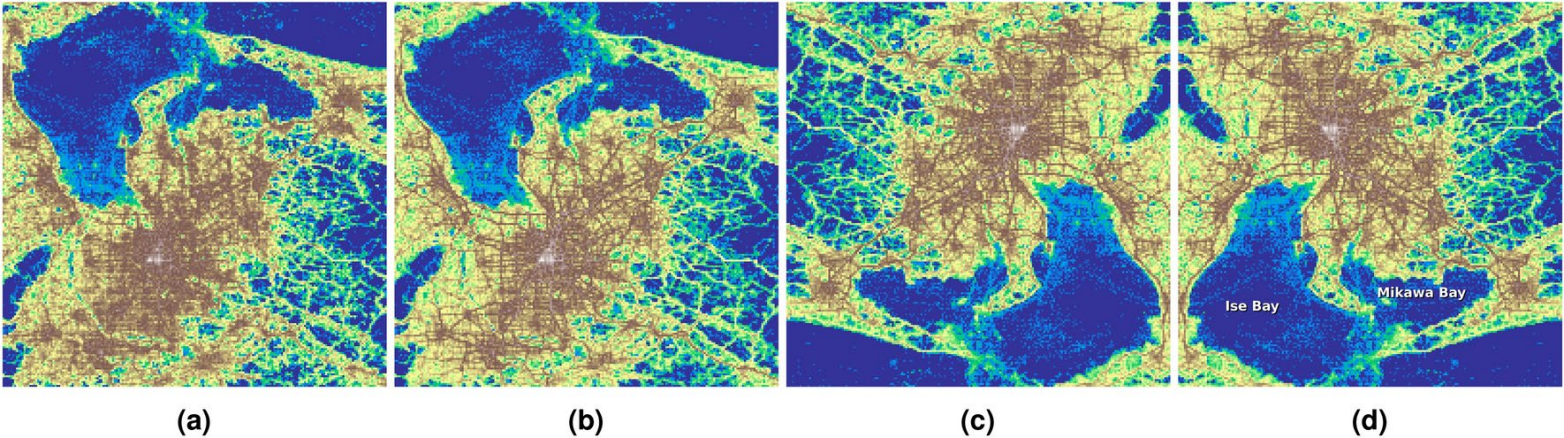
The heatmaps showing the number of activity records (**a**) and the number of unique users (**b**) in the observation area. The latter is rotated by $180^{\circ }$ (**c**) and flipped horizontally (**d**).

First, Figure 6a and 6b show a 2-dimensional histogram of the number of pings and the number of observed unique users over the 75 days on a log scale, respectively which are analogous to the Figure 6of the data description paper^11^. The urban area is clearly visible with the road network, especially when the number of unique users is plotted (Figure 6b). The city silhouette from the heatmap is then compared with the cities from Figure 5, it is clear that the observation area is the Nagoya metropolitan area, but the figure is transformed. After rotating the image by $180^{\circ }$ (Figure 6c) and flipping it horizontally (Figure 6d) (or flipping it vertically), it becomes clear that the large low-activity areas at the top of the untransformed heatmaps (Figure 6b) are actually Ise Bay and Mikawa Bay (denoted in Figure 6d).

**Fig. 7:**
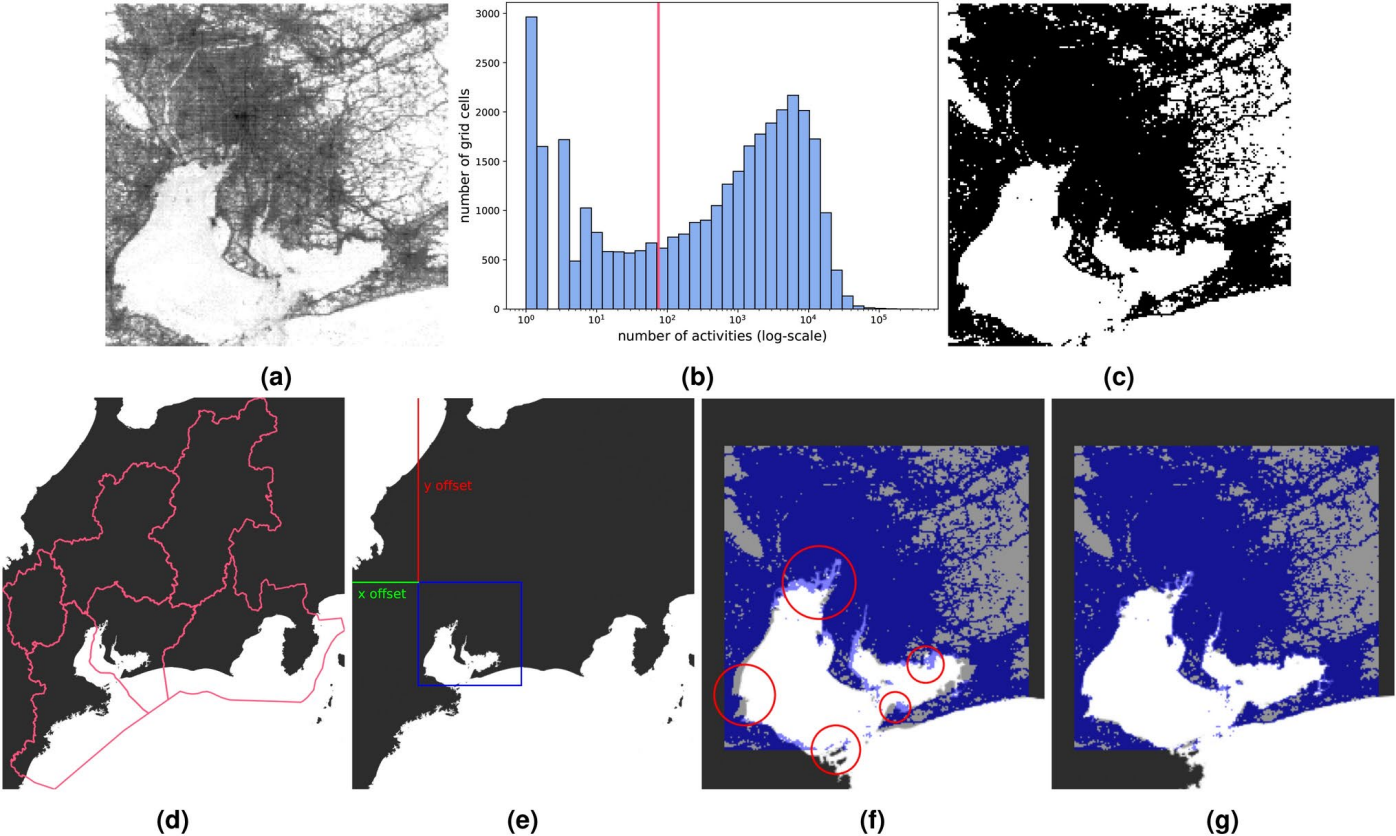
The first step of the template matching process is to create a template. Starting with the grayscale heatmap of the activity (**a**), then the histogram of the activity records is determined (**b**). The threshold value is selected (horizontal line), then pixels below the threshold are set to white, pixels above the threshold are set to black (**c**). The land in the bounding box of the six selected prefectures (**d**), which serves as the input for the template matching (where **c** is the template). The result location of the template matching is illustrated in **e**. The template is displayed over the map (**f**), and some obvious mismatches are highlighted. After the correction (stretching) the overlapping shows perfect match (**g**).

The next problem is to find the exact location of the spatial grid that was used to discretize the spatial location of the mobility data. The basic idea is to fit the heatmap to a proportional map as an image, taking advantage of the fact that Ise Bay and Mikawa Bay have a peculiar shape. The plot of the log-scale activity (ping) heatmap using a grayscale palette is shown in Figure 7a, Figure 7b shows the activity histogram, as well as the selected threshold (75). Note that, since the threshold was applied directly to the data, the grayscale palette is not required, it is just shown for better understanding. Figure 7c shows the binary image as a result of the thresholding. This process made the coastal area crisp by removing the activity at the ferry lines, although there are low-activity rural lands at the western part of the region, that may appear as water in the binary image. More details are presented about the impact of the threshold value on the template matching process in Section S2 of the supplementary information.

Six prefectures were downloaded from OSM via OSM-Boundaries^36^: Aichi, Gifu, Mie, Nagano, Shiga, Shizuoka. The coastline of the Japanese islands was also needed, the data is from OSM, as pre-processed land polygons from^37^, and the main islands of Japan were extracted manually. Figure 7d shows of a bounding box of the selected six prefectures, which will serve as the input for the template matching stage, which is a method to find the location of a template image in a larger image^38^. The land geometry should be scaled to be proportional to the template, the observation area, which is a 200 by 200 grid. The grid is plotted as a 200 by 200 pixel image, with each cell representing a 500 by 500 meter area. Consequently, the bounding box of the selected six prefectures area should be plotted with a scale of 1 pixel = 500 meters.

Figure 7e shows the result of the template matching, which determines the *x* and the *y* offset of the template image. In this case, the thresholded activity heatmap (Figure 7c). The resulting area of the land (blue square in Figure 7e) and its surroundings are shown in Figure 7f with the semitransparent template.

Figure 7f also highlights some obvious mismatches, although the matching is relatively correct. It appears that the template has been squeezed vertically and horizontally stretched. Since the template should not be transformed to maintain the conversion rate of ‘1 pixel equals to 500 meters’, the land image was transformed instead. To determine the exact horizontal and vertical stretching ratio, the background and the template were loaded into an image manipulation program, and the layer was manually stretched by trial and error. The final values are 10% horizontal stretching and 10% vertical shrinking. Figure 7g shows the same comparison after these transformations were applied to the background image, and the match is perfect.

The template matching returns the *x* and *y* offsets of the template (Figure 7e) in the input image, which was transformed and scaled to be proportional to the template. Applying the inverse transformations to the offset coordinates determines the top-left point of the grid in the geographic coordinate system. Using the reference point, a 200 by 200 grid should be generated with a 500-meter width and height.

The cell coordinates start from the upper left corner according to the description paper^11^ and increase to right and down. There are two possible ways to join the challenge data with the reconstructed grid: (i) transform the coordinates in the mobility data and start coordinates as described, or (ii) assign transformed coordinate properties to the grid geometry and keep the mobility data as provided. This work uses the second option. The transformed grid coordinates are visualized in Figure 1a.

## Supplementary Information

Supplementary Information.

## Data Availability

The ‘YJMob100K’ the ‘Weeplaces’, and the Helsinki data sets are also available on Zenodo^25,27^:, and^39^, respectively. The ‘Dallas–Fort Worth metroplex’ data set is available from Dryad^28^. The census data was downloaded from the Portal Site of Official Statistics of Japan website (https://www.e-stat.go.jp/): Population, Households, Sex, Age and Marital Status, *Population Census 2020*, Table 1-1.

## Code availability

The code to reproduce this work is available on GitHub: https://github.com/pintergreg/reverse-engineering-YJMob100K-grid
