# Supplementary material for: Revealing urban area from mobile positioning data
Source: Sci Rep. 2024 Dec 28;14:30948. doi: 10.1038/s41598-024-82006-5 (PMC11681113; doi:10.1038/s41598-024-82006-5)
Supplement: Supplementary file 1 — Supplementary Information. [file 41598_2024_82006_MOESM1_ESM.pdf]

Supplementary information for
Revealing urban area from mobile positioning data

Gergő Pintér [1,*]

[1]ANETI Lab, Corvinus Institute for Advanced Studies, Corvinus University of Budapest, Budapest, 1093, Hungary
[*]Corresponding author: gergo.pinter@uni-corvinus.hu

S1 Adding noise to the location data

It has been proposed in the literature that the addition of random noise to the mobility data can enhance individual privacy [1]. The "Weeplaces" dataset [2], which was utilized in the Toronto and London experiments, contains precise GPS coordinates. To assess the noise resilience of the urban landscape identification, Gaussian noise was added to the longitude and latitude of each record. The Gaussian noise was applied with multiple standard deviation (spread or width) values, including 250 m, 500 m, 1 km, and 2 km. Subsequently, the template matching process was executed with the aforementioned four cell sizes (0.5 km, 1 km, 2 km, and 4 km) for both Toronto and London. The application of Gaussian noise has the effect of distributing the activities (or appearances) of the mobility data in a given circle.

From the perspective of computer vision literature, the noise behaves like a blurring effect, which removes the details, while preserving the rough shape of the city, as long as the "blur" is applied within reasonable scales (without the intent of completely destroying the data). On the other hand, some face recognition algorithms are capable of achieving a high recognition rate on low-resolution images (128×128 pixels and below) [3, 4], thus the template should contain sufficient detail for identifying the city. Figure S1a illustrates the blurring effect on the template. The cells represented by black contained activity before and after the blurring, blue cells contains activity because it was relocated by the noise effect, and red cells lost their activity due to the activity relocation. As expected, the area covered by activities increased, but the shape of the urban area remained roughly the same.

Figure S1b and S1c present a summary of the identification results for Toronto and London, respectively. The values of the matrices indicate the Euclidean distance between the upper-left corner of the observation area and the result of the template matching in meters. Template matching was performed for varying cell sizes (columns) and standard deviations (rows) of the Gaussian noise. While some mismatches were observed in the case of Toronto, these were rare and marginal in the case of London. It should be noted that the cell size also represents the identification resolution.

It is possible that the noise may significantly enhance individual privacy (this was not tested in the present study), but it has little effect on the recognition of urban areas. Note that the evaluation of noise addition is not without limitations. This approach applies a naive method for adding noise to the locations and may decrease the usability of the mobility data. The addition of noise was implemented for the purpose of evaluating the urban landscape recognition. The integrity and meaningfulness of the mobility data were not a primary concern in this process.

Furthermore, the map area is still limited to a relatively modest neighborhood of the metropolitan area in question, and a few units of difference in the localization is tolerated. As with the YJMob100K data, the main text's validation demonstrates that even with a few units of difference, the grid is correctly located. Also note that, due to the sparsity of the "Weeplaces" data set the threshold for the data set is zero; every cell is considered black if it contains activity and white otherwise. For more details about the threshold selection, see Section S2.

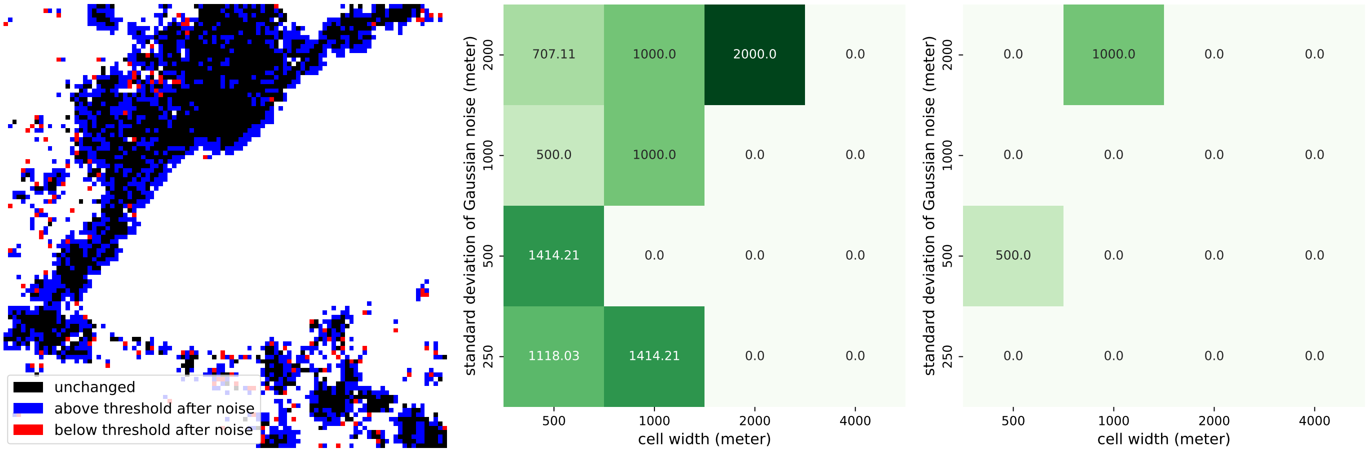

(a) (b) (c)

Figure S1: The impact of Gaussian noise (1 km standard deviation) on the Toronto data set (**a**) is illustrated using a 1 km by 1 km grid. The summary matrix presents the results of template matching in the context of the Gaussian noise, specifically in the case of Toronto (**b**) and London (**c**). The values displayed in the matrix represent the Euclidean distance between the upper-left corner of the observation area and the result of the template matching operation, expressed in meters. The template matching operation was performed for a variety of cell sizes and standard deviations of the Gaussian noise.

Table S1: Comparison of the template matching results with different thresholds. The template anchor points (upper left corner) are compared to the value used in the main text (75) and the difference is expressed in cells (500 meters).

threshold	25	50	100	200	300	400	500	600	700	800	900	1000	2000	3000	4000	5000
x	0	0	0	0	0	0	0	0	0	0	-228	-228	65	61	-387	-387
y	0	0	0	1	1	1	1	1	1	1	44	45	150	149	78	79

S2 Threshold

This section offers a more detailed examination of the impact of the threshold on the template matching process. Thresholding is applied to the activity heatmap to maximize the contrast between the high activity cells and the low activity cells. The threshold represents the value that determines the boundary between the low and high activity (in this case). Figure S2 illustrates a comparison of templates with varying threshold values from 25 to 5000 in alignment with Figure 7c of the main text. However, the accompanying histograms are included in the figures. They also show how much detail of the coastline is lost as the threshold is increased. At a threshold of 2000 (Figure S2g), the Chita Peninsula and the Atsumi Peninsula (annotated in Figure S2a) disappear almost completely. Table S1 shows a comparison of the accuracy of the template matching results using templates generated with different thresholds. Up to threshold 800 the template matching gives good results, above this threshold the observation area is not located correctly. From 200 to 800 there is one unit of difference in the y-axis, but this is considered acceptable because it is only one pixel difference in terms of the template matching. From 900 the differences are significant and the template matching has clearly failed.

Template matching does not require binary images to work. A binary template was used to simplify the matching problem. Figure S3a shows a heatmap using an inverse grayscale palette, where high activity is light and low activity is dark, which can also be used as a template. The map can be rendered using, for example, CyclOSM tiles, which also show the city labels (in Japanese), and then converted to grayscale. The grayscale conversion can be done by several methods. Figure S3b shows a luminance preserving method, while Figure S3b shows a minimum based method, where the minimum of the three color component values is used for the pixel. The difference is also illustrated by histograms (Figure S3d). Note that the histogram is cropped, so the actual frequency (about 480,000) of the value representing the water is omitted. The minimum-based grayscale results

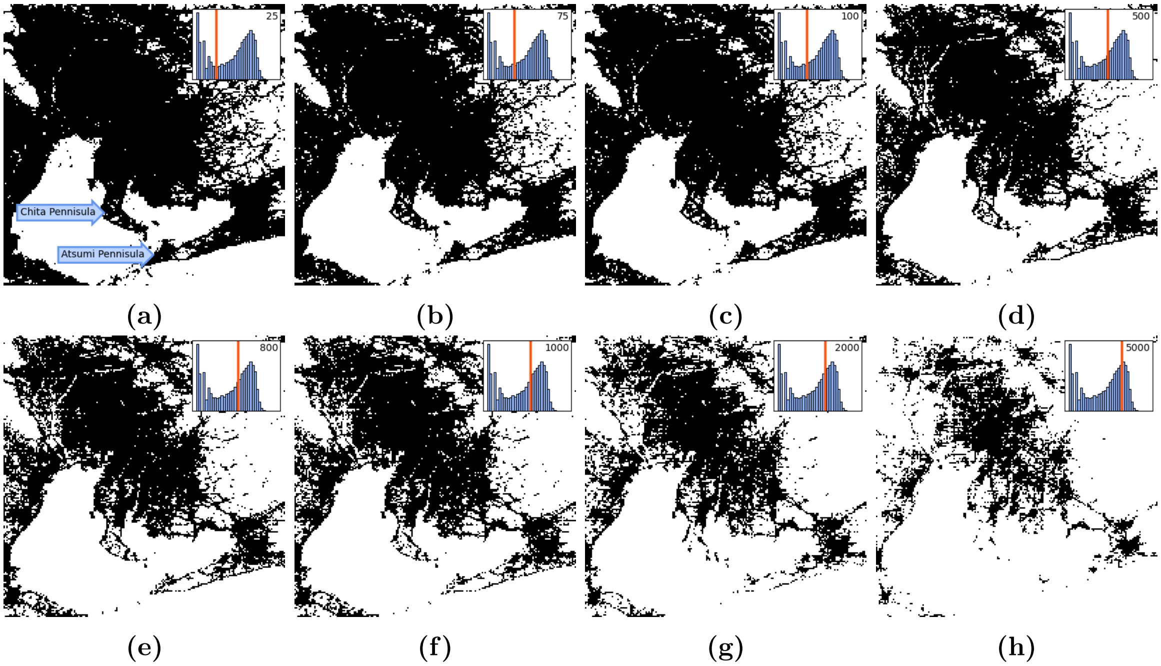

Figure S2: Templates with different threshold values of 25, 75, 100, 500, 800, 1000, 2000, and 5000 (**a**, **b**, **c**, **d**, **f**, **g**, and **h**, respectively). The figures also show the activity histogram with the threshold value (as in Figure 7c of the main text).

in darker colors and in this case more contrast between the inhabited and uninhabited location. This subtle difference in the input image can be enough to determine the result of the template matching, depending on the matching method used. For this scenario the `cv2.TM_SQDIFF_NORMED` method was used. For the binary templates the `cv2.TM_SQDIFF` method performs better because it can tolerate higher thresholds (shown in Table S1). The `cv2.TM_SQDIFF_NORMED` method cannot find the binary templates generated with a threshold higher than 400.

However, it is important to emphasize that this study is not about template matching applied to mobility data. The technical differences between the matching methods are beyond the scope of this study. The purpose of this supplementary section is to provide more insight into the methods applied. The presented identification method is one possible solution for revealing the urban area from mobile positioning data, but it is certainly not the only one. Once the activity heatmap and the map use the same coordinate system the correct relative position of the template on the map can be found manually.

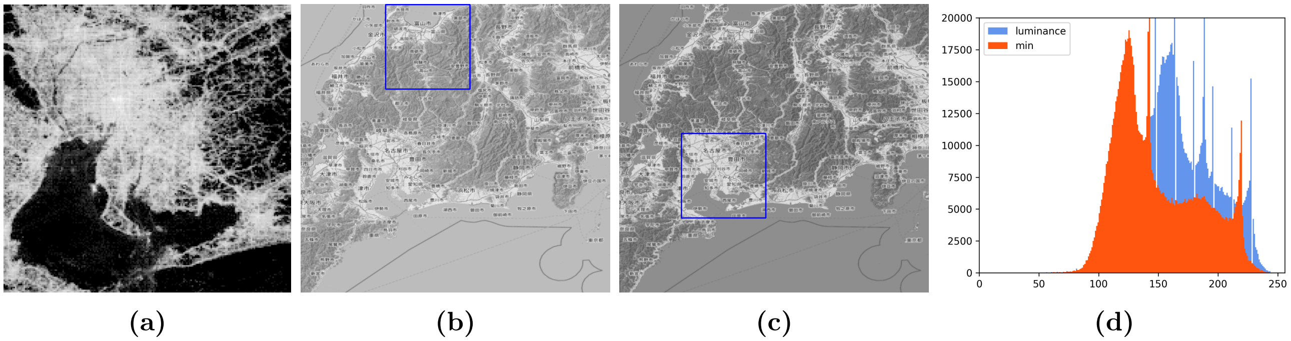

Figure S3: Example of template matching with grayscale template and maps. The template (**a**) is inverse grayscale, where the high activity locations are light, and the low activity locations are dark. The map is rendered using CyclOSM tiles and converted to grayscale using luminance-preserving (**b**), and minimum-based(**c**) methods. The difference is also visualized by histograms (**d**). The identified locations are also denoted in (**b**) and (**c**), showing that the matching was successful only in the case of the latter one, with more contrast.

References

[1] Gergely Acs, Szilvia Lestyán, and Gergely Biczók. "Privacy of Aggregated Mobility Data". In: *Encyclopedia of Cryptography, Security and Privacy*. Springer, 2021, pp. 1–5.

[2] Zexun Chen. *Spatiotemporal checkins with social connections*. Mar. 2022. DOI: `https://doi.org/10.5281/zenodo.6369319`. URL: `https://doi.org/10.5281/zenodo.6369319`.

[3] Pei Li et al. "Face recognition in low quality images: A survey". In: *arXiv preprint arXiv:1805.11519* (2018).

[4] Xiang Xu, Wan-Quan Liu, and Ling Li. "Low resolution face recognition in surveillance systems". In: *Journal of Computer and Communications* 2 (2014), pp. 70–77.
